# Emergency All-Arterial Anaortic Off-Pump Coronary Artery Bypass Grafting for Acute Coronary Syndrome in a 52-Year-Old Patient with a Childhood History of Kawasaki Disease

**DOI:** 10.70352/scrj.cr.26-0176

**Published:** 2026-06-16

**Authors:** Hiroyuki Saito, Takeshi Hiramatsu, Noriyasu Masuda

**Affiliations:** Department of Cardiovascular Surgery, Yachiyo Medical Center, Tokyo Women’s Medical University, Yachiyo, Chiba, Japan

**Keywords:** Kawasaki disease, acute coronary syndrome, off-pump coronary artery bypass grafting, anaortic technique, all-arterial grafting, transitional care

## Abstract

**INTRODUCTION:**

Kawasaki disease (KD) is a leading cause of acquired heart disease in children. Although intravenous immunoglobulin (IVIG) therapy significantly reduces coronary complications, long-term management of these patients as they transition into adulthood remains a clinical challenge. We report a rare case of a 52-year-old patient from the first generation of IVIG-treated survivors of KD who required emergency surgical intervention for acute coronary syndrome (ACS).

**CASE PRESENTATION:**

A 52-year-old man presented with persistent chest pain and dyspnea. He had been diagnosed with KD at the age of 5 and was treated with IVIG. Although he was monitored for coronary aneurysms until the age of 18, he had been lost to medical follow-up for 34 years and did not receive antiplatelet therapy during this period. Coronary angiography revealed complete occlusion of the proximal left anterior descending and right coronary arteries, with calcified aneurysms at both coronary orifices. Emergency off-pump coronary artery bypass grafting (OPCABG) was performed using an all-arterial anaortic technique. The left internal thoracic artery was grafted to the left anterior descending artery, and the right gastroepiploic artery was grafted to the posterior descending artery. The calcified aneurysms were left intact to avoid complex reconstruction requiring cardiopulmonary bypass. The postoperative course was uneventful, and coronary CT confirmed excellent graft patency. The patient was discharged on POD 14.

**CONCLUSIONS:**

As early generations of patients with KD reach their 50s, the combination of pre-existing vascular vulnerability and age-related atherosclerosis may increase the risk of acute coronary events. This case demonstrates that anaortic OPCABG using all-arterial grafts is a safe and effective strategy for managing ACS in this specific clinical scenario. It also highlights the need for standardized transitional care protocols to ensure continuous cardiovascular monitoring from childhood to adulthood.

## Abbreviations

ACS
: acute coronary syndrome
CABG
: coronary artery bypass grafting
CPB
: cardiopulmonary bypass
IVIG
: intravenous immunoglobulin
KD
: Kawasaki disease
LITA
: left internal thoracic artery
OPCAB
: off-pump coronary artery bypass grafting
PDA
: posterior descending artery
RGEA
: right gastroepiploic artery
TTFM
: transit-time flow meter

## INTRODUCTION

KD was first identified in 1967 and has become the leading cause of acquired heart disease in children in developed nations.^1)^ The introduction of IVIG therapy in the early 1980s has markedly reduced the incidence of coronary aneurysms and subsequent arterial complications.^1,2)^ However, as the first generation of patients treated with IVIG has reached their 50s, a new clinical challenge has emerged: the intersection between KD-related vascular sequelae and age-related atherosclerosis.^3)^

While the existing literature extensively covers pediatric management, data on the surgical management of adult survivors with late-stage KD complications are still limited.^4,5)^ These patients often harbor long-term vascular endothelial dysfunction and chronic low-grade inflammation, making them susceptible to acute coronary events, even when their childhood aneurysms were classified as low risk.^5)^ This report describes a rare case in which emergency anaortic OPCAB was used to treat ACS in a 52-year-old man who had been lost to follow-up for more than 3 decades after initial KD treatment.

## CASE PRESENTATION

A 52-year-old man presented to our institution with persistent chest pain and dyspnea. He had a history of KD at the age of 5 years and was treated with IVIG. Although he was monitored for coronary aneurysms (<8 mm) until the age of 18, he discontinued medical follow-up after high school and did not receive further antiplatelet or anticoagulant therapy thereafter. His cardiovascular risk factors included dyslipidemia (low-density lipoprotein-cholesterol 165 mg/dL) and a 32 pack-year smoking history.

Laboratory evaluation revealed elevated high-sensitivity troponin I levels (133.50 ng/mL). Electrocardiography showed no ST-segment elevation; however, transthoracic echocardiography demonstrated a left ventricular ejection fraction of 44% with anteroseptal wall dyssynergy. No significant valvular disease was identified. Emergency coronary angiography revealed complete occlusion of the proximal left anterior descending and right coronary arteries, while the circumflex artery showed no significant stenosis (**Fig. 1**). The PDA was supplied by collateral circulation originating from the left coronary artery (Rentrop grade 2). The SYNTAX score was calculated as 36.5, indicating high lesion complexity. Substantial coronary aneurysms with circumferential eggshell calcification (maximum short-axis diameter, 8 mm) were observed at both coronary orifices, together with intracoronary collaterals (**Fig. 2**).

**Fig. 1:**
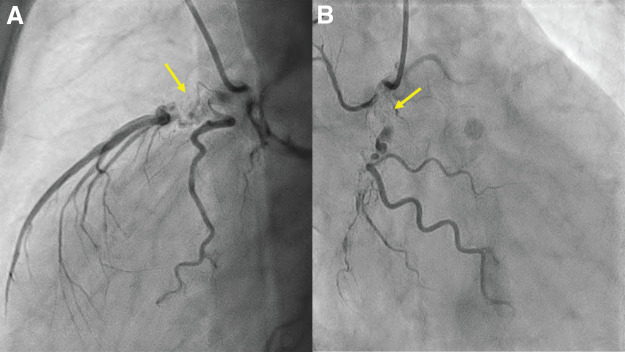
Preoperative coronary angiogram. (**A**) LCA: The proximal left anterior descending artery was completely occluded (arrow) along with the presence of intracoronary collaterals. The circumflex artery showed no significant stenosis. (**B**) The proximal right coronary artery was completely occluded (arrow) with distal CTO. CTO, chronic total occlusion; LCA, left coronary artery

**Fig. 2:**
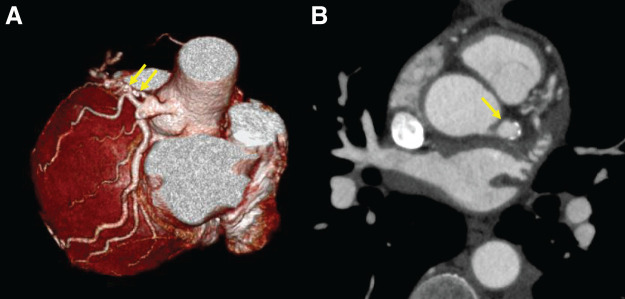
Contrast-enhanced CT. (**A**) 3D image: The proximal left anterior descending artery was occluded (arrows). (**B**) The coronary aneurysm of the LCA reached a maximum short-axis diameter of 8 mm and exhibited dense, circumferential (360°-degree) “eggshell” calcification (arrow). LCA, left coronary artery

Because of the complex stenotic anatomy and aneurysmal changes, percutaneous coronary intervention was not recommended. Emergency double OPCAB was performed using an all-arterial anaortic technique. The LITA was anastomosed to the left anterior descending artery, and the RGEA to the PDA. The calcified aneurysms were left intact to avoid CPB and complex reconstruction. Intraoperative graft flow assessed using a TTFM was excellent, with a mean flow rate of 35 mL/min in the LITA graft and 18 mL/min in the RGEA graft. Although pulsatility index and diastolic filling values were not measured due to device limitations, the patient’s hemodynamic stability and postoperative CT confirmed graft patency. The operative time was 226 min, and the patient was extubated 155 min postoperatively. After 3 days in the ICU, the postoperative course was uneventful. Coronary CT confirmed graft patency (**Fig. 3**), and the patient was discharged on POD 14.

**Fig. 3:**
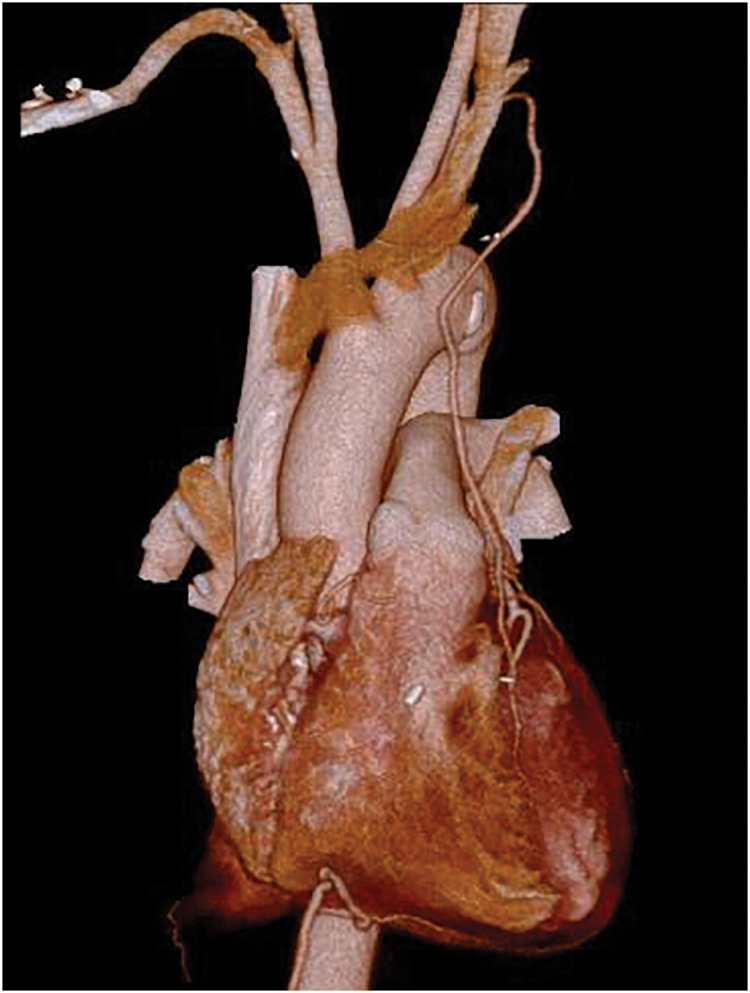
Postoperative contrast-enhanced CT. 3D image revealed graft patency.

## DISCUSSION

The surgical findings in this case provide valuable insights into the pathophysiology of KD in adults. The coronary artery walls at the anastomosis sites appeared normal despite the proximal aneurysmal changes. However, the arteries were relatively narrow, likely because of chronic ischemia and reduced flow during development. These findings underscore the synergistic effect of pre-existing KD-related vascular vulnerability and lifestyle-related factors such as smoking and dyslipidemia in accelerating atherosclerosis.^5)^

A major practical challenge highlighted by this case is loss to follow-up. Patients who remain asymptomatic after adolescence often stop seeking medical care, unaware that they still require antiplatelet therapy and strict risk factor management.^2,6)^ As the first IVIG-treated generation reaches the age at which metabolic syndrome becomes more prevalent, an increase in similar ACS cases may be observed. Establishing standardized transitional care protocols is crucial to ensuring that these patients remain within the healthcare system. Specific strategies include the use of structured transfer documents summarizing coronary artery morphology and intervention history, and the implementation of joint clinics in which pediatric and adult cardiologists collaborate.^1,7)^ According to the JCS 2020 guidelines, regular risk assessment and lifestyle modification are recommended even for patients who are asymptomatic during adolescence.^2)^ These models aim to prevent the loss to follow-up observed in our case and ensure long-term cardiovascular surveillance.

From a surgical perspective, an all-arterial anaortic OPCAB strategy was particularly advantageous in this case. In contrast to conventional CABG, which requires aortic manipulation or CPB, our anaortic technique minimized the risk of embolic events from potentially calcified vessels and reduced surgical stress in this hemodynamically unstable patient. However, it is important to note that the optimal surgical strategy should be individualized. Although previous reports have described surgical interventions for adult KD primarily in elective settings using on-pump CABG to ensure precise anastomosis and address complex target lesions,^4,8)^ our case demonstrates the feasibility and benefits of an all-arterial anaortic OPCAB approach specifically in an emergency ACS setting with a high embolic risk.^1)^ Indeed, conventional on-pump CABG remains the established standard for elective adult KD survivors—especially when complex aneurysmal reconstruction or concomitant valvular surgery is required; nevertheless, our experience suggests that the anaortic OPCAB strategy can be a valuable option for selected patients presenting with acute hemodynamic instability and extensive cardiovascular calcification. Furthermore, arterial grafts such as the LITA and RGEA are particularly beneficial for long-term patency in this relatively young patient population, offering superior durability compared with venous grafts.^8)^

## CONCLUSIONS

We successfully treated a 52-year-old survivor of KD with ACS using emergency anaortic OPCAB. This case emphasizes that survivors of KD require lifelong cardiovascular surveillance to prevent late-stage thrombotic events. Establishing standardized transitional care protocols is crucial to ensuring that these patients remain within the healthcare system from childhood to adulthood.

## DECLARATIONS

### Availability of data and materials

The data supporting this study’s findings are available from the corresponding author upon reasonable request.
